# Impact of frailty on the performance of the National Early Warning Score 2 to predict poor outcome in patients hospitalised due to COVID-19

**DOI:** 10.1186/s12877-023-03842-0

**Published:** 2023-03-08

**Authors:** Peter Selmer Rønningen, Marte Meyer Walle-Hansen, Håkon Ihle-Hansen, Elizabeth Lyster Andersen, Arnljot Tveit, Marius Myrstad

**Affiliations:** 1 Department of Medical Research, Bærum Hospital, Vestre Viken Hospital Trust, Post Box 800, 3004 Drammen, Norway; 2 Department of Internal Medicine, Bærum Hospital, Vestre Viken Hospital Trust, Gjettum, Norway; 3 Institute of Clinical Medicine, Faculty of Medicine, University of Oslo, Oslo, Norway

**Keywords:** Frailty, NEWS-2, Prognosis, COVID-19

## Abstract

**Background:**

The National Early Warning Score 2 (NEWS2) is a scoring tool predictive of poor outcome in hospitalised patients. Older patients with COVID-19 have increased risk of poor outcome, but it is not known if frailty may impact the predictive performance of NEWS2. We aimed to investigate the impact of frailty on the performance of NEWS2 to predict in-hospital mortality in patients hospitalised due to COVID-19.

**Methods:**

We included all patients admitted to a non-university Norwegian hospital due to COVID-19 from 9 March 2020 until 31 December 2021. NEWS2 was scored based on the first vital signs recorded upon hospital admission. Frailty was defined as a Clinical Frailty Scale score ≥ 4. The performance of a NEWS2 score ≥ 5 to predict in-hospital mortality was assessed with sensitivity, specificity and area under the receiver operating characteristic curve (AUROC) according to frailty status.

**Results:**

Out of 412 patients, 70 were aged ≥ 65 years and with frailty. They presented less frequently with respiratory symptoms, and more often with acute functional decline or new-onset confusion. In-hospital mortality was 6% in patients without frailty, and 26% in patients with frailty. NEWS2 predicted in-hospital mortality with a sensitivity of 86%, 95% confidence interval (CI) 64%-97% and AUROC 0.73, 95% CI 0.65–0.81 in patients without frailty. In older patients with frailty, sensitivity was 61%, 95% CI 36%-83% and AUROC 0.61, 95% CI 0.48–0.75.

**Conclusion:**

A single NEWS2 score at hospital admission performed poorly to predict in-hospital mortality in patients with frailty and COVID-19 and should be used with caution in this patient group.

**Graphical Abstract:**

Graphical abstract summing up study design, results and conclusion

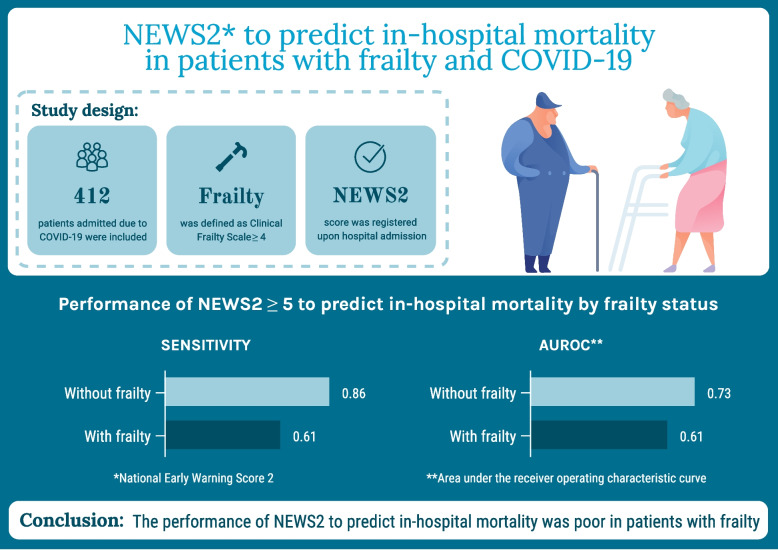

**Supplementary Information:**

The online version contains supplementary material available at 10.1186/s12877-023-03842-0.

## Background

Frailty is characterised by cumulative loss of functional capacity and reserves, and is highly prevalent among older hospitalised patients. Frailty associates with increased vulnerability to external stressors, such as acute illness [1]. Clinical manifestations of critical coronavirus disease 2019 (COVID-19) include respiratory failure and prolonged need of ventilator supportive treatment [2, 3]. Older individuals are particularly vulnerable to infection with severe acute respiratory syndrome coronavirus 2 (SARS-CoV-2), with increased risk of critical COVID-19 and higher COVID-19 mortality [4, 5]. Frailty is identified as an additional risk factor for poor outcome [6, 7].

The pandemic has challenged the capacity of hospitals and intensive care units. Rapid assessment tools could identify patients in need of escalated clinical care, and aid decision making and prioritisation in acute care settings. Consequently, clinical tools to assess risk of deterioration and poor outcome at an early stage, have been subject to evaluation. The National Early Warning Score 2 (NEWS2) has shown promising results for this purpose [8]. In an early report, we found that a single NEWS2 scoring performed in the Emergency Department upon hospital admission could predict in-hospital mortality and critical disease in patients hospitalised due to COVID-19 with high sensitivity and specificity, and was superior to other commonly used clinical risk scores [9]. NEWS2 has been recommended to assist clinical judgement in patients hospitalised with COVID-19 for assessment of a patient’s risk of deterioration [10], and is widely used.

Older persons often present with atypical symptoms when hospitalised due to COVID-19 [11]. The complexity of acutely ill older persons may interfere with the diagnostic evaluation and complicate risk assessment and clinical decision making [12], but there is limited knowledge on whether frailty impacts the performance of NEWS2 to predict poor outcome in older hospitalised patients. The aim of this study was to evaluate the performance of a single NEWS2 scoring in the Emergency Department upon hospital admission to predict in-hospital mortality and critical disease according to frailty status in patients acutely admitted to hospital due to COVID-19.

## Methods

In this cohort study, we included all patients admitted to Bærum Hospital due to COVID-19 from the start of the pandemic until 31 December 2021. Bærum Hospital is a non-university hospital serving approximately 190,000 inhabitants in the capital region of Norway, with more than 11,000 acute admissions per year. COVID-19 was diagnosed by qualitative detection of nucleic acid from SARS-CoV-2, using real-time polymerase chain reaction from nasal swab tests.

We excluded SARS-CoV-2-positive patients admitted for other reasons and for whom COVID-19 had no relation to the admission. If there was doubt to the question if COVID-19 and concomitant factors could have contributed to the admission, the patient were included or excluded after consensus between the first and last author. All data were collected retrospectively from patient records.

We used the Norwegian translation of the Clinical Frailty Scale (CFS) to characterise the overall level of fitness and frailty of the patients, based on retrospective review of patient records. CFS is a 9-level scale assessing functional capacity, based on independency in activities of daily living and health status two weeks prior to the onset of acute illness [13]. While a CFS score of 1–3 is used to describe people who are very fit, fit and well managing, a score of 4 describes people with very mild frailty. A score of 5–8 describes people with mild, moderate, severe and very severe frailty, who are in need of assistance in daily activities. Terminally ill people are scored with 9. CFS is easily applied, little time-consuming and thus feasible for use in the acute setting. Already on 20 March 2020, the National Institute for Health and Care Excellence recommended the use of CFS in patients with COVID-19 [10]. Retrospective scoring of CFS in clinical practice research in older hospitalised patients, based on already collected information about functional capacity, has been validated previously, with acceptable results [14]. For patients staying at nursing homes or receiving home care services, information on functional status with detailed descriptions of domains of activities of daily living is transmitted electronically to our hospital upon admission. Thus, health records contained sufficient information to determine CFS. Four physicians (PSR, MMWW, HIH, MM) with clinical and academic experience took part in the CFS assessment. As CFS is validated only in persons aged 65 years and older, we excluded patients aged younger than 65 years with a CFS score of 4 or higher in the current analyses.

NEWS2 is a clinical scoring tool of six physiological parameters: respiratory rate, oxygen saturation, systolic blood pressure, pulse rate, body temperature, and level of consciousness or new-onset confusion. Each parameter is scored with 0 to 3 points, and 2 points are added for patients requiring supplementary oxygen treatment. The scoring tool was developed to improve detection of clinical deterioration in acutely ill patients and a score of 5 or 6 and higher, should trigger urgent response by a clinician [15]. NEWS2 is recommended for use in patients hospitalised due to COVID-19 and in routine use at Bærum Hospital. In the current study, NEWS2 scores were calculated based on the first vital signs recorded in the Emergency Department upon hospital admission, documented in charts or electronic records. We used a score of 5 or higher as cut-off.

We defined the main outcome in-hospital mortality as death related or unrelated to COVID-19 during the hospital stay. Since patients with frailty more frequently have treatment level limitations, critical disease defined as death during the hospital stay or treatment at the intensive care unit, was assessed as secondary outcome.

Symptoms of COVID-19 prior to admission were self-reported or reported by a next-of-kin and registered based on documentation in patient records. We used the Charlson Comorbidity Index to characterise patients in terms of chronic comorbidities such as heart failure, chronic kidney disease, chronic obstructive pulmonary disease, and malignancy [16]. Body mass index was calculated based on patient height and weight registered during the hospital stay. Smoking habits were self-reported at admission.

### Statistical methods

Continuous variables are presented as the median and interquartile range. Categorical variables are presented as numbers and percentages. The performance of NEWS2 to predict in-hospital mortality and critical disease, was assessed by sensitivity, specificity, positive predictive value, negative predictive value, and the area under the receiver operating characteristic curve (AUROC). The corresponding 95% confidence interval (95% CI) was calculated. We assessed the performance in patients with and without frailty with a CFS score cut-off ≥ 4. Since a CFS score of 4 represents very mild frailty, we performed secondary analyses with CFS score 5 (mild frailty) as cut-off. To evaluate the impact of age group on our results, we performed sensitivity analyses after exclusion of patients younger than 65 years [17]. Data were analysed with STATA 17 (StataCorp, College Station, Texas).

### Ethical considerations

All methods were carried out in accordance with relevant guidelines and regulations. This quality study was approved by the Data Protection Officer, Vestre Viken Hospital Trust, n-3004 Drammen, Norway (ref: 20/02772–17). Since only routine clinical data were collected from the electronic health records, the Data Protection Officer, Vestre Viken Hospital Trust, n-3004 Drammen, Norway (ref: 20/02772–17) waived the requirement for informed consent. A letter with information about the study was sent by post to all patients, allowing the patient to withdraw their data. The study complies with the Declaration of Helsinki.

## Results

**Fig. 1:**
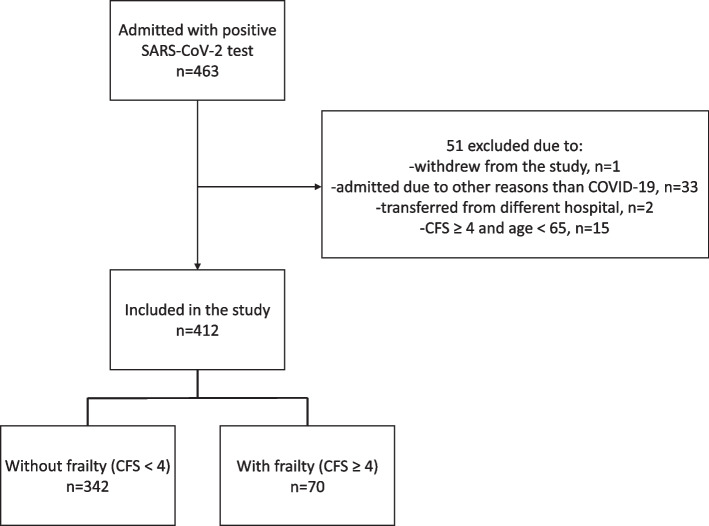
Flow chart of study population. SARS-CoV-2 indicates Severe Acute Respiratory Syndrome Coronavirus 2; COVID-19, Coronavirus Disease 2019; CFS, Clinical Frailty Scale

A total of 463 patients positive for SARS-CoV-2 were admitted to the hospital from 9 March 2020 until 31 December 2021. Of these, two were transferred from a different hospital and lacked data on vital signs on admittance, and one withdrew from the study. In agreement between the first and last author, 33 patients were considered admitted for reasons without any relation to the finding of SARS-CoV-2 and excluded from the analyses. Moreover, we excluded 15 patients with a CFS score ≥ 4 and aged < 65 years. Figure 1 presents inclusion of participants to the study.

Out of the total study population of 412 patients, 70 (17%) were aged 65 years and older and scored with CFS ≥ 4. Table 1 shows characteristics of the study population according to frailty status. Patients with frailty seemed to have more comorbidities and lower body mass index than patients without frailty.

**Table 1:** Study population characteristics by frailty status

	Without frailty (CFS < 4) *n* = 342	With frailty (CFS ≥ 4) *n* = 70
Age, years	53.0 (45.0–65.0)	81.0 (77.0–88.0)
Male sex, n (%)	218 (63.7%)	35 (50.0%)
Body mass index, (kg/m2)	26.6 (24.1–29.9)	24.1 (21.5–27.8)
Charlson Comorbidity Index	1.0 (0.0–3.0)	6.0 (5.0–7.0)
Hypertension, n (%)	67 (19.6%)	33 (47.1%)
Diabetes mellitus, n (%)	48 (14.0%)	19 (27.1%)
Cardiac conditions, n (%)	32 (9.4%)	31 (44.3%)
Chronic kidney disease, n (%)	4 (1.2%)	18 (25.7%)
COPD, n (%)	10 (2.9%)	15 (21.4%)
Asthma, n (%)	39 (11.4%)	5 (7.1%)
Malignancy, n (%)	16 (4.7%)	14 (20.0%)
Current or previous smoking, n (%)	96 (28.7%)	28 (40.6%)

Values are median (interquartile range) or numbers (percentages). CFS indicates Clinical Frailty Scale; *COPD* Chronic obstructive pulmonary disease. There were 47 missing values for body mass index and 8 for smoking status

Table 2 shows symptoms and NEWS2 score upon hospital admission according to frailty status. Patients with frailty seemed to have slightly shorter duration of symptoms prior to hospitalisation, less frequent respiratory symptoms and fever, but more frequent new-onset confusion and acute functional decline. There were no major differences in inflammatory markers. Except from a high occurrence of acute confusion among patients with frailty, NEWS2 subscores and total score seemed to be comparable regardless of frailty status. In Supplementary Table 1, NEWS2 scoring including vital signs as continuous variables are presented according to frailty status and in-hospital mortality. Among patients who died during the hospital stay, those with frailty seemed to less frequently have hypoxemia and requirement of oxygen supplementation, and more frequently tachycardia and acute confusion compared to patients without frailty. The proportion with fever seemed to be comparable.

**Table 2:** Symptoms, vital signs, and NEWS2 by frailty status

	Without frailty (CFS < 4) *n* = 342	With frailty (CFS ≥ 4) *n* = 70
Admitted from nursing home, n (%)	3 (0.9%)	10 (14.3%)
Duration of symptoms prior to admission, days	8.0 (5.0–10.0)	4.0 (2.0–7.0)
*Symptoms prior to admission*		
Cough, n (%)	202 (59.1%)	29 (41.4%)
Dyspnoea, n (%)	227 (66.4%)	35 (50.0%)
Fever, n (%)	257 (75.1%)	33 (47.1%)
Reduced general condition, n (%)	240 (70.2%)	44 (62.9%)
New confusion, n (%)	12 (3.5%)	17 (24.3%)
Acute functional decline, n (%)	15 (4.4%)	38 (54.3%)
*Laboratory findings*		
CRP at admission, mg/L	56.0 (25.0–112.0)	52.0 (20.0–78.0)
Leukocytes at admission, 10E9/L	5.7 (4.4–7.6)	6.4 (5.0–8.9)
*NEWS2 scoring*		
Respiratory rate ≥ 22/minute, n (%)	139 (40.6%)	28 (40.0%)
Oxygen saturation ≤ 93%, n (%)	110 (32.2%)	26 (37.1%)
Oxygen supplement, n (%)	132 (38.6%)	26 (37.1%)
Systolic blood pressure < 100 mm Hg, n (%)	12 (3.5%)	3 (4.3%)
Heart rate > 90 beats/minute, n (%)	133 (38.9%)	23 (32.9%)
Acute confusion, n (%)	8 (2.3%)	12 (17.1%)
Body temperature > 38 C, n (%)	144 (42.1%)	26 (37.1%)
NEWS2 score	4.0 (1.0–6.0)	4.0 (2.0–7.0)
NEWS2 score ≥ 5, n (%)	147 (43.0%)	31 (44.3%)

Values are median (interquartile range) or numbers (percentages). NEWS2 indicates National Early Warning Score version 2; *CFS* Clinical Frailty Scale, *CRP* C-reactive protein. There were 6 missing values for duration of symptoms, 2 missing values for CRP and Leukocytes at admission

The median length of hospital stay was 8 (interquartile range: 4–13) days in the total study population and was comparable regardless of frailty status. Total in-hospital mortality was 9%, 26% in patients with frailty and 6% in patients without frailty. Critical disease occurred in 30% of patients with frailty and in 26% of patients without frailty.

The performance of a NEWS2 score ≥ 5 to predict poor outcome according to frailty status is presented in Table 3. In patients with frailty, NEWS2 exhibited poor performance to predict in-hospital mortality, with a sensitivity of 61% (95% CI 36%-83%) and AUROC 0.61 (95% CI 0.48–0.75). The predictive performance of NEWS2 to predict critical disease showed similar estimates.

**Table 3:** Performance of NEWS2 ≥ 5 to predict poor outcome by frailty status

	Sensitivity (95% CI)	Specificity (95% CI)	PPV (95% CI)	NPV (95% CI)	AUROC (95% CI)
Prediction of in-hospital mortality					
Without frailty, (CFS < 4)	85.7% (63.7%-97.0%)	59.8% (54.2%-65.2%)	12.2% (7.4%-18.7%)	98.5% (95.6%-99.7%)	0.73 (0.65-0.81)
With frailty, (CFS ≥ 4)	61.1% (35.7%-82.7%)	61.5% (47.0%-74.7%)	35.5% (19.2%-54.6%)	82.1% (66.5%-92.5%)	0.61 (0.48-0.75)
Prediction of critical disease					
Without frailty, (CFS < 4)	78.4% (68.4%-86.5%)	69.3% (63.2%-74.9%)	46.9% (38.7%-55.3%)	90.3% (85.2%-94.0%)	0.74 (0.69-0.79)
With frailty, (CFS ≥ 4)	61.9% (38.4%-81.9%)	63.3% (48.3%-76.6%)	41.9% (24.5%-60.9%)	79.5% (63.5%-90.7%)	0.63 (0.50-0.75)

NEWS2 indicates National Early Warning Score version 2; *CI* Confidence interval, *PPV* Positive predictive value, *NPV* Negative predictive value, *AUROC* Area under the receiver operating characteristic curve, *CFS* Clinical Frailty Scale; critical disease defined as death or treatment at the intensive care unit

NEWS2 score predicted in-hospital mortality in the total study population with a sensitivity of 74% (95% CI 58%-87%) and AUROC of 0.67 (95% CI 0.60–0.75). For prediction of critical disease, sensitivity was 75% (95% CI 66%-83%) and AUROC 0.72 (95% CI 0.67–0.77). The study design and main results are summarised in the graphical abstract.

Out of the total patient population without frailty, there were 93 patients aged 65 years or older. Of these, 14 (15%) died and 26 (28%) experienced critical disease. After exclusion of patients < 65 years, the median age in patients without frailty was 74 years (interquartile range 70–79) and the median CCI was 4 (interquartile range 3–5). The predictive performance of NEWS2 in these patients was comparable to the total population without frailty, except for the positive predictive value of mortality that was increased (Supplementary Table 2).

We also performed the analyses with CFS cut-off at ≥ 5 showing poor predictive performance of NEWS2 in patients with frailty. Sensitivity was 57% (95% CI 29%-82%), and AUROC 0.55 (95% CI 0.39–0.72) to predict in-hospital mortality.

## Discussion

This cohort study among patients hospitalised due to COVID-19 suggests that the performance of NEWS2 score to predict poor outcome is poor in patients with frailty. To our best knowledge, this is the first study to investigate the impact of frailty on the performance of NEWS2 to predict poor outcome. Thus, the study adds clinically relevant knowledge regarding the use of NEWS2 in this setting. It suggests that the complexity of acutely ill older persons with frailty, including atypical presentation of symptoms, should be taken into consideration during diagnostic evaluation.

In acute care, such as in the Emergency Department, rapid assessment of risk of deterioration is central to aid decision making and prioritisation. For the purpose of identifying patients at need of escalated care, the sensitivity of a test is considered the key parameter. In the current study, sensitivity in patients without frailty at 86% is acceptable, whereas a sensitivity at 61% in patients with frailty questions the utility of NEWS2 in this patient group. For the outcome of in-hospital mortality, positive predictive value was higher in patients with frailty, but this likely reflects the increased occurrence of death among these patients rather than the performance of the test.

In line with previous studies, we found that older patients with frailty and COVID-19 often presented with atypical symptoms, such as new-onset confusion [18]. Previous studies have demonstrated that delirium is highly prevalent and associated with increased mortality among patients hospitalised due to COVID-19 [19].

In another cohort study of hospitalised COVID-19 patients, adding vulnerability to NEWS2 improved the predictive accuracy [20]. Although with a different approach than in our study, these studies together suggest that physiologic parameters alone are not sufficient for accurate prediction of poor outcome in older patients hospitalised due to COVID-19.

The reason why the performance of NEWS2 was poor in patients with frailty remains unanswered, but our data may provide some insights. Patients with frailty who died seemed to less frequently have hypoxemia and requirement of oxygen supplementation, but more frequently tachycardia and acute confusion. A possible weakness of NEWS2 is that the scoring of oxygen supplementation is binary, and thus a higher demand of oxygen will not necessarily lead to a higher NEWS2 score if hypoxemia is adequately treated. Patients with frailty are often characterised by comorbidity, and concomitant conditions could have contributed to atypical presentation of symptoms and clinical signs.

In concordance with previous observations, our study demonstrates a high in-hospital mortality in older patients with frailty. Although studies have reported a gradual increase in mortality with increasing CFS-score [21], a cut-off value may be desirable for clinical purposes. We decided to use a CFS score of 4 or higher to define frailty in our main analysis. With stricter definition of frailty, and a CFS cut-off at 5 or higher, NEWS2 seemed even less useful for the purpose of interest.

The current study reminds of the limitations of clinical scoring tools. It remains undisputable that NEWS2 and other tools should be used with caution and only as supplement to a more comprehensive diagnostic evaluation and frailty assessment, particularly in older patients. Prospective studies should evaluate the value of clinical scoring tools across different medical conditions among older patients with frailty, and with clinically relevant endpoints.

### Strengths and limitations

A main strength of this study is that we consecutively included all patients admitted to a local Norwegian hospital due to COVID-19 during 34 months from the very beginning of the pandemic. The study cohort comprises a relatively large patient population in one of the largest non-university hospitals in Norway, and includes patients from all waves of the COVID-19 pandemic, improving the external validity of the results. Furthermore, NEWS2 scores are based on complete data systematically registered as part of the clinical routine in our hospital.

A limitation of the study is that we assessed frailty retrospectively, based on patient records. Previous studies, however, have demonstrated acceptable validity of retrospective scoring of CFS in older hospitalised patients [14]. Furthermore, a new version of CFS was introduced during the study period, with slightly different descriptions of the categories.

The single NEWS2 scoring upon hospital admission is another limitation, as patients with critical disease may have had more advanced disease already at this point of time. On the other hand, the study demonstrates the value of a single measurement of vital signs early after admission to identify patients in need of increased monitoring, a finding highly relevant in times of a pandemic challenging the capacity of many hospitals worldwide. There were relatively few patients with frailty and accordingly, there is uncertainty in the estimates. The study lacked power to statistically assess differences in the performance of NEWS2 in subgroups. As expected, patients without frailty were younger than patients with frailty. However, it was beyond the scope of this study to assess whether it was older age per se or frailty that limited the predictive performance of NEWS2 in this study population. Lack of data on vaccinations status is a limitation, as this may have had an effect on the disease course in an unknown number of patients after vaccination started around 1 January 2021. Treatment, such as Dexamethason, which was routinely used in patients with respiratory failure from July 2020, may also have affected the estimates. These temporal changes to vaccination status, treatment strategies and emergence of new virus variants questions whether the findings can be generalised to current-day practice.

## Conclusion

The performance of a single NEWS2 scoring upon hospital admission to predict in-hospital mortality and critical disease was poor in patients with frailty. Patients with frailty often presented atypical symptoms. NEWS2 and other clinical scoring tools should be used with caution in acutely ill patients with frailty.

## Supplementary Information

**Additional file 1: Supplementary Table 1.** NEWS2 subscores and total scores by frailty status and in-hospital mortality. Supplementary **Table 2. **Performance of NEWS2 ≥ 5 to predict poor outcome by frailty status in patients aged 65 years or over.

## Data Availability

The datasets generated and analysed during the current study are not publicly available because the approval by the data protection officer does not allow for such publication, but are available from senior author Marius Myrstad on reasonable request.

## Abbreviations

COVID-19: Coronavirus disease 2019
SARS-CoV-2: Severe acute respiratory syndrome coronavirus 2
NEWS2: National Early Warning Score 2
CFS: Clinical Frailty Scale
AUROC: Area under the receiver operating characteristic curve
95% CI: 95% Confidence interval

## References

[1] Clegg A, Young J, Iliffe S, Rikkert MO, Rockwood K (2013). Frailty in elderly people. The Lancet.

[2] Richardson S, Hirsch JS, Narasimhan M, Crawford JM, McGinn T, Davidson KW (2020). the Northwell C-RC, Barnaby DP, Becker LB, Chelico JD *et al*: Presenting Characteristics, Comorbidities, and Outcomes Among 5700 Patients Hospitalized With COVID-19 in the New York City Area. JAMA.

[3] Wu Z, McGoogan JM (2020). Characteristics of and Important Lessons From the Coronavirus Disease 2019 (COVID-19) Outbreak in China: Summary of a Report of 72 314 Cases From the Chinese Center for Disease Control and Prevention. JAMA.

[4] Becerra-Munoz VM, Nunez-Gil IJ, Eid CM, Garcia Aguado M, Romero R, Huang J, Mulet A, Ugo F, Rametta F, Liebetrau C (2021). Clinical profile and predictors of in-hospital mortality among older patients hospitalised for COVID-19. Age Ageing.

[5] Reilev M, Kristensen KB, Pottegård A, Lund LC, Hallas J, Ernst MT, Christiansen CF, Sørensen HT, Johansen NB, Brun NC (2020). Characteristics and predictors of hospitalization and death in the first 11 122 cases with a positive RT-PCR test for SARS-CoV-2 in Denmark: a nationwide cohort. Int J Epidemiol.

[6] Blomaard LC, van der Linden CMJ, van der Bol JM, Jansen SWM, Polinder-Bos HA, Willems HC, Festen J, Barten DG, Borgers AJ, Bos JC (2021). Frailty is associated with in-hospital mortality in older hospitalised COVID-19 patients in the Netherlands: the COVID-OLD study. Age Ageing.

[7] Rottler M, Ocskay K, Sipos Z, Görbe A, Virág M, Hegyi P, Molnár T, Erőss B, Leiner T, Molnár Z (2022). Clinical Frailty Scale (CFS) indicated frailty is associated with increased in-hospital and 30-day mortality in COVID-19 patients: a systematic review and meta-analysis. Ann Intensive Care.

[8] Zhang K, Zhang X, Ding W, Xuan N, Tian B, Huang T, Zhang Z, Cui W, Huang H, Zhang G (2021). The Prognostic Accuracy of National Early Warning Score 2 on Predicting Clinical Deterioration for Patients With COVID-19: A Systematic Review and Meta-Analysis. Front Med (Lausanne).

[9] Myrstad M, Ihle-Hansen H, Tveita AA, Andersen EL, Nygard S, Tveit A, Berge T (2020). National Early Warning Score 2 (NEWS2) on admission predicts severe disease and in-hospital mortality from Covid-19 - a prospective cohort study. Scand J Trauma Resusc Emerg Med.

[10] COVID-19 rapid guideline: managing COVID-19. https://www.nice.org.uk/guidance/ng191. Accessed 23 June 2022. 34181371

[11] Poco PCE, Aliberti MJR, Dias MB, Takahashi SF, Leonel FC, Altona M, Venys AL, Shin-Ike IA, Garcia BA, Sumita LH (2021). Divergent: Age, Frailty, and Atypical Presentations of COVID-19 in Hospitalized Patients. J Gerontol A Biol Sci Med Sci.

[12] Wester AL, Dunlop O, Melby KK, Dahle UR, Wyller TB (2013). Age-related differences in symptoms, diagnosis and prognosis of bacteremia. BMC Infect Dis.

[13] Rockwood K, Song X, MacKnight C, Bergman H, Hogan DB, McDowell I, Mitnitski A (2005). A global clinical measure of fitness and frailty in elderly people. CMAJ.

[14] Stille K, Temmel N, Hepp J, Herget-Rosenthal S (2020). Validation of the Clinical Frailty Scale for retrospective use in acute care. Eur Geriatr Med.

[15] National Early Warning Score (NEWS) 2: Standardising the assessment of acute-illness severity in the NHS. Updated report of a working party. https://www.rcplondon.ac.uk/projects/outputs/national-early-warning-score-news-2. Accessed 23 June 2022.

[16] Charlson ME, Pompei P, Ales KL, MacKenzie CR (1987). A new method of classifying prognostic comorbidity in longitudinal studies: development and validation. J Chronic Dis.

[17] Myrstad M, Ronningen PS, Tveita A, Ronning EJ, Erno PE, Andersen EL, et al. Three waves of COVID-19 in a Norwegian local hospital. Tidsskr Nor Laegeforen. 2022;141(2). 10.4045/tidsskr.21.0750. 10.4045/tidsskr.21.0750 35107949

[18] Marziliano A, Burns E, Chauhan L, Liu Y, Makhnevich A, Zhang M, Carney MT, Dbeis Y, Lindvall C, Qiu M (2022). Patient Factors and Hospital Outcomes Associated With Atypical Presentation in Hospitalized Older Adults With COVID-19 During the First Surge of the Pandemic. J Gerontol A Biol Sci Med Sci.

[19] Shao SC, Lai CC, Chen YH, Chen YC, Hung MJ, Liao SC (2021). Prevalence, incidence and mortality of delirium in patients with COVID-19: a systematic review and meta-analysis. Age Ageing.

[20] Aliberti MJR, Covinsky KE, Garcez FB, Smith AK, Curiati PK, Lee SJ, Dias MB, Melo VJD, Rego-Junior OFD, Richinho VP (2021). A fuller picture of COVID-19 prognosis: the added value of vulnerability measures to predict mortality in hospitalised older adults. Age Ageing.

[21] Aw D, Woodrow L, Ogliari G, Harwood R (2020). Association of frailty with mortality in older inpatients with Covid-19: a cohort study. Age Ageing.

