# Supplementary material for: Impact of frailty on the performance of the National Early Warning Score 2 to predict poor outcome in patients hospitalised due to COVID-19
Source: BMC Geriatr. 2023 Mar 8;23:134. doi: 10.1186/s12877-023-03842-0 (PMC9994778; doi:10.1186/s12877-023-03842-0)
Supplement: Supplementary file 1 — Additional file 1: Supplementary Table 1. NEWS2 subscores and total scores by frailty status and in-hospital mortality. Supplementary Table 2. Performance of NEWS2 ≥ 5 to predict poor outcome by frailty status in patients aged 65 years or over. [file 12877_2023_3842_MOESM1_ESM.docx]

SUPPLEMENTARY MATERIAL

| **Supplementary table 1.** NEWS2 subscores and total scores by frailty status and in-hospital mortality | | | | |
| --- | --- | --- | --- | --- |
|  | Without frailty (CFS < 4) n=342 | | With frailty (CFS ≥ 4) n=70 | |
|  | Discharged alive n=321 | Died during hospital stay n=21 | Discharged alive n=52 | Died during hospital stay n=18 |
| Respiratory rate, breaths/minute | 20.0 (18.0-24.0) | 22.0 (18.0-27.0) | 20.0 (17.0-22.0) | 23.5 (20.0-29.0) |
| Respiratory rate ≥ 22/minute, n (%) | 127 (39.6%) | 12 (57.1%) | 17 (32.7%) | 11 (61.1%) |
| Oxygen saturation, % | 95.0 (93.0-97.0) | 89.0 (84.0-92.0) | 95.0 (92.0-96.0) | 93.5 (91.0-95.0) |
| Oxygen saturation ≤ 93%, n (%) | 94 (29.3%) | 16 (76.2%) | 17 (32.7%) | 9 (50.0%) |
| Oxygen supplement, n (%) | 115 (35.8%) | 17 (81.0%) | 17 (32.7%) | 9 (50.0%) |
| Systolic blood pressure, mm Hg | 127.0 (118.0-141.0) | 126.0 (120.0-137.0) | 130.5 (117.5-156.0) | 134.5 (112.0-152.0) |
| Systolic blood pressure < 100 mm Hg, n (%) | 12 (3.7%) | 0 (0.0%) | 2 (3.8%) | 1 (5.6%) |
| Heart rate, beats/minute | 87.0 (75.0-97.0) | 86.0 (72.0-96.0) | 80.0 (69.0-90.0) | 94.0 (81.0-100.0) |
| Heart rate > 90 beats/minute, n (%) | 125 (38.9%) | 8 (38.1%) | 12 (23.1%) | 11 (61.1%) |
| Acute confusion, n (%) | 5 (1.6%) | 3 (14.3%) | 8 (15.4%) | 4 (22.2%) |
| Body temperature, C | 37.8 (37.2-38.5) | 37.9 (37.6-38.1) | 37.7 (37.0-38.3) | 38.0 (37.6-38.5) |
| Body temperature > 38 C, n (%) | 137 (42.7%) | 7 (33.3%) | 19 (36.5%) | 7 (38.9%) |
| NEWS2 score | 4.0 (1.0-6.0) | 7.0 (6.0-9.0) | 3.0 (2.0-6.0) | 6.0 (4.0-9.0) |
| NEWS2 score ≥ 5, n (%) | 129 (40.2%) | 18 (85.7%) | 20 (38.5%) | 11 (61.1%) |
| Values are median (interquartile range) or numbers (percentages). NEWS2 indicates National Early Warning Score version 2; CFS, Clinical Frailty Scale | | | | |

| **Supplementary table 2.** Performance of NEWS2 ≥ 5 to predict poor outcome by frailty status in patients aged 65 years or over | | | | | |
| --- | --- | --- | --- | --- | --- |
| *Prediction of in-hospital mortality* | | | | | |
|  | Sensitivity (95% CI) | Specificity (95% CI) | PPV (95% CI) | NPV (95% CI) | AUROC (95% CI) |
| Without frailty, (CFS < 4) | 85.7% (57.2%-98.2%) | 62.0% (50.4%-72.7%) | 28.6% (15.7%-44.6%) | 96.1% (86.5%-99.5%) | 0.74 (0.63-0.85) |
| With frailty,  (CFS ≥ 4) | 61.1% (35.7%-82.7%) | 61.5% (47.0%-74.7%) | 35.5% (19.2%-54.6%) | 82.1% (66.5%-92.5%) | 0.61 (0.48-0.75) |
| *Prediction of critical disease* | | | | | |
|  | Sensitivity (95% CI) | Specificity (95% CI) | PPV (95% CI) | NPV (95% CI) | AUROC (95% CI) |
| Without frailty, (CFS < 4) | 76.9% (56.4%-91.0%) | 67.2% (54.6%-78.2%) | 47.6% (32.0%-63.6%) | 88.2% (76.1%-95.6%) | 0.72 (0.62-0.82) |
| With frailty,  (CFS ≥ 4) | 61.9% (38.4%-81.9%) | 63.3% (48.3%-76.6%) | 41.9% (24.5%-60.9%) | 79.5% (63.5%-90.7%) | 0.63 (0.50-0.75) |
| NEWS2 indicates National Early Warning Score version 2; CI, confidence interval; PPV, positive predictive value, NPV, negative predictive value; AUROC, area under the receiver operating characteristic curve; CFS, Clinical Frailty Scale; critical disease, death or treatment at the intensive care unit | | | | | |
